# Evaluation of Nanaerobic Digestion as a Mechanism to Explain Surplus Methane Production in Animal Rumina and Engineered Digesters

**DOI:** 10.1021/acs.est.2c07813

**Published:** 2023-08-11

**Authors:** Zhuoying Wu, Duc Nguyen, Shilva Shrestha, Lutgarde Raskin, Samir Kumar Khanal, Po-Heng Lee

**Affiliations:** † Department of Civil and Environmental Engineering, Imperial College London, London SW7 2AZ, United Kingdom; ‡ Department of Molecular Biosciences and Bioengineering, University of Hawai’i at Ma̅noa, Honolulu 96822, Hawaii, United States; § Department of Civil and Environmental Engineering, University of Michigan, 1351 Beal Avenue, 107 EWRE Building, Ann Arbor 48109, Michigan, United States; ∥ Joint BioEnergy Institute, Emeryville, California 94608, United States; ⊥ Biological Systems and Engineering Division, Lawrence Berkeley National Laboratory, Berkeley, California 94720, United States; # Shanghai Shaanxi Coal Hi-tech Research Institute Co., Ltd., Shanghai 201613, China; ∇ The Lyell Centre, Heriot-Watt University, Edinburgh EH14 4AS, United Kingdom

**Keywords:** nanaerobe, biogas, anaerobic digestion, nanaerobic respiration, cytochrome *bd* oxidase, oxygen, microaeration, rumen

## Abstract

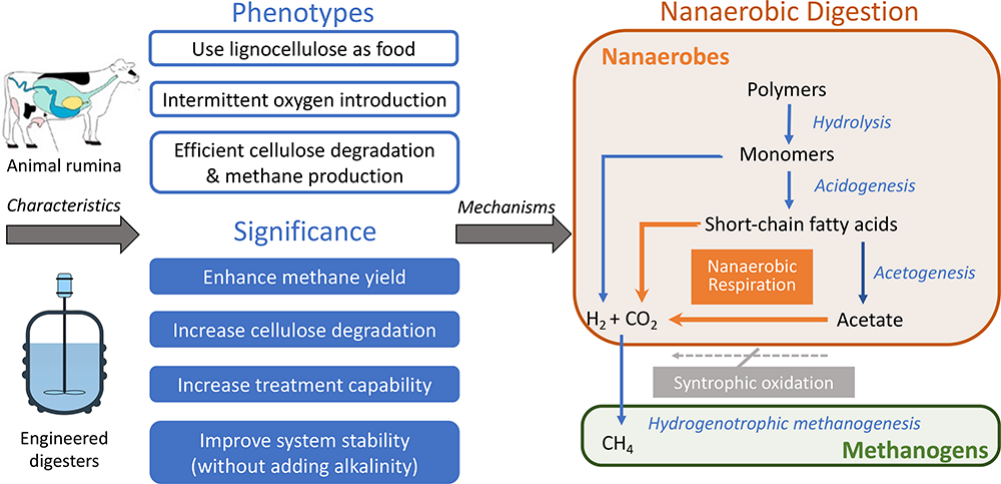

Nanaerobes are a newly described class of microorganisms that use a unique cytochrome *bd* oxidase to achieve nanaerobic respiration at <2 μM dissolved oxygen (∼1% of atmospheric oxygen) but are not viable above this value due to the lack of other terminal oxidases. Although sharing an overlapping ecological niche with methanogenic archaea, the role of nanaerobes in methanogenic systems has not been studied so far. To explore their occurrence and significance, we re-analyzed published meta-omic datasets from animal rumina and waste-to-energy digesters, including conventional anaerobic digesters and anaerobic digesters with ultra-low oxygenation. Results show that animal rumina share broad similarities in the microbial community and system performance with oxygenated digesters, rather than with conventional anaerobic digesters, implying that trace levels of oxygen drive the efficient digestion in ruminants. The rumen system serves as an ideal model for the newly named nanaerobic digestion, as it relies on the synergistic co-occurrence of nanaerobes and methanogens for methane yield enhancement. The most abundant ruminal bacterial family *Prevotellacea*e contains many nanaerobes, which perform not only anaerobic fermentation but also nanaerobic respiration using cytochrome *bd* oxidase. These nanaerobes generally accompany hydrogenotrophic methanogens to constitute a thermodynamically and physiologically consistent framework for efficient methane generation. Our findings provide new insights into ruminal methane emissions and strategies to enhance methane generation from biomass.

## Abstract 2

We demonstrated that a unique ecological niche (dissolved oxygen <2 μM) was associated with the co-occurrence of methanogens and nanaerobes, which we suggest result in enhanced methane production in animal rumina and engineered digesters. We propose to call this process nanaerobic digestion.

## 1 Introduction

Methane emission by ruminant animals contributes significantly to climate change,^1^ whereas methane production in waste-to-energy anaerobic digestion systems is vital for a sustainable future.^2^ Biomethane is generally considered to be produced by four anaerobic microbial groups, including hydrolyzers, acidogens, acetogens, and methanogens. These anaerobes are commonly believed to be viable below 1% of atmospheric oxygen, corresponding to approximately 2 μM dissolved oxygen (DO) at 25 °C.^3−5^ This DO level also fits the growth pattern of most facultative bacteria with heme-copper oxidases (types A, B, and C, see Table S1).^6−11^ Below 2 μM DO, they switch from aerobic respiration to anaerobic fermentation for energy production. Nanaerobes, which encode single cytochrome *bd* oxidase, were proposed as a new class of microorganisms in 2004.^12^ Due to the extremely high oxygen affinity of cytochrome *bd* oxidase, nanaerobes can respire aerobically at DO levels as low as 3 nM, which is two to three orders of magnitude lower than previously observed for aerobes.^13^ Moreover, since they lack other oxidases present in conventional facultative bacteria, nanaerobes are not viable above 2 μM DO,^12^ and their respiratory lifestyle has been termed nanaerobic respiration.^14^ Therefore, a special DO niche (0 < DO <2 μM) may exist for both nanaerobes and obligate anaerobes (including methanogens), creating a goldilocks paradigm for the synergistic partnership between nanaerobic respiration and anaerobic methanogenesis, referred to as nanaerobic digestion in this study. To the best of our knowledge, the occurrence and environmental significance of nanaerobes in methanogenic systems have not been explored before.

Some micro-oxygenated anaerobic digestion studies have provided clues about the role of nanaerobes in methane generation. It was reported that injection of a small amount of air or oxygen into traditional anaerobic digesters significantly enhanced organic matter degradation, alleviated volatile fatty acid (VFA) accumulation, and increased methane production.^15,16^ The phenomenon has long been attributed to the participation of the aforementioned normal facultative bacteria.^15,16^ However, an ideal DO dosing regimen that would balance aerobic and anaerobic metabolisms has not been established. Nguyen et al.^17^ and Wu et al.^18^ began to notice a positive relationship between nanaerobe involvement and methane enhancement in lignocellulosic biomass digestion with an intermittent oxidation–reduction potential (ORP)-controlled micro-aeration system, referred to as ORP-controlled micro-oxygenated digester. The cytochrome *bd*-encoding nanaerobe *Proteiniphilum* sp. was highly enriched in each of their experimental triplicates when ORP values were set at 25 mV above the anaerobic baseline ORP of ∼−500 mV (below nanomolar DO), increasing the methane yield by 3.1-fold (from 22.9 ± 3.7 to 70.8 ± 3.7 mL CH_4_/g volatile solids (VS)) and improving the VS reduction by 2.3-fold (from 20.6 ± 2.7 to 47.3 ± 2.7%) at an organic loading rate as high as 5 g VS/L/day.^17,18^ These results indicated that this enhanced performance may be due to the co-occurrence of cytochrome *bd*-induced nanaerobic respiration and the traditional four anaerobic digestion steps that facilitated methane production. However, due to the operational complexity (unexpected oxygen) and data incompleteness (absence of microbial activity data) in these engineered systems, the concept of nanaerobic digestion, an enhancement of methane production driven by nanaerobes, was not fully established.

Ruminant digestion is not only efficient in lignocellulose degradation to produce VFAs for animal growth but also emits substantial amounts of methane, contributing to global warming.^19^ Studies of rumina have focused only on the above four anaerobic microbial groups and have left numerous unanswered questions. There have been no effective strategies to maintain animal productivity while reducing methane emission.^20,21^ Meanwhile, attempts to simulate rumen digestion by inoculating anaerobic bioreactors with rumen content have proven unsuccessful for long-term operation due to the washout of rumen microbial populations.^22−24^ Thus, there are undiscovered factors that shape the rumen microbiome. Ultra-small amounts of oxygen likely reach the intestinal tract of various animals,^25,26^ including the rumen.^27^ It was reported that DO levels of ∼1 μM had been detected *in situ* in rumina of cows, sheep, and goats shortly after feeding,^21^ suggesting that oxygen is introduced during feeding. Trace amounts of oxygen may also be introduced during rumination, a process involving regurgitation of previously ingested food back to the mouth for a second mastication. Therefore, the effectiveness of rumen digestion may also be linked with the process of nanaerobic digestion, where nanaerobic respiration may be induced by the intermittent injection of nanomolar levels of oxygen during rumination. Although there are difficulties of precise comparison between rumen performances due to their varied sizes, they may represent perfect scenarios for the evolution of nanaerobe prevalence as ultra-low oxygen tension in animal rumina.

Based on the above rationale, this study aimed to demonstrate the novel concept of nanaerobic digestion, where the long overlooked but critical contributors, nanaerobes, perform nanaerobic respiration through the use of cytochrome *bd* oxidase and concurrently participate in the traditional four-step anaerobic digestion for methane generation. Considering the three striking similarities between ORP-controlled oxygenated lignocellulosic biomass digesters and animal rumina, (1) both are fed with lignocellulosic substrates, (2) both are subjected to intermittent exposure to oxygen, and (3) both are efficient methane producers,^17,18,20,21^ these two ecosystems may be appropriate to illustrate the concept. However, digester studies are designed to compare performance yet make it difficult to avoid variation in microbiome dynamics associated with the occasional external oxygen intrusion. In contrast, due to the delicate variation in oxygen flux, rumina allow the investigation of microbiome reproducibility but do not exhibit much difference in performance. In this study, we mainly focused on the responses of ruminal microbial communities (oxygen-consuming bacteria and cooperative methanogenic archaea) to the intermittent low oxygen delivery through rumination. To demonstrate and evaluate the prevalence of nanaerobes and their respiratory activity in methanogenic systems, we specifically assessed the diversity and expression of bacterial terminal oxidase genes responsible for oxygen consumption, as well as their affiliated taxonomy ranks, using metagenomic and metatranscriptomic approaches. We also elucidated the most likely metabolic pathway under such low oxygen condition through thermodynamics analysis to reveal the nature of the methanogen population present in nanaerobic digestion. Finally, since enhanced methane production has been well demonstrated in ORP-controlled and other micro-oxygenated digesters,^15−18^ the rumen samples were retrospectively compared with digester samples in terms of microbial community and performance metrics. Such analyses facilitate in establishing an unprecedented correlation between methane yield and abundance of nanaerobes in two typical model systems.

## 2 Materials and Methods

### 2.1 Sample Selection

Metagenomic and metatranscriptomic datasets were used to mine information on the abundance and activity of nanaerobic bacteria, and oxygen and methane concentrations were used to evaluate their relationship (Table 1). For rumina, recent studies mainly focused on collecting metagenomic data to investigate metabolic pathways associated with atmospheric methane emission. There are, however, limited studies focusing on metatranscriptomic data. Meanwhile, due to the low oxygen delivery during rumination, rumen studies typically lack such direct information about DO levels, but DO levels might be proportional with the rumination frequency and duration.^28^ In addition, due to the microbiome complexity in animal rumina, it would be better to utilize deep sequencing data to pinpoint an unbiased profile of nanaerobe prevalence. Therefore, four ultra-deep-sequenced rumen samples (∼90 Gb of metagenomic data and the corresponding metatranscriptomic data) from two high- and two low-methane-emitting sheep, which had been classified by Shi et al.,^29^ were selected for our study. Considering that cattle are the main ruminant animals contributing to methane emissions, we included metagenomic and corresponding metatranscriptomic datasets for four cattle samples (no methane information was available for these samples).^30^ Three additional cattle samples with methane and metagenomic datasets were also studied (no metatranscriptomic data were available for these samples).^31^ Relevant digester studies usually focused more on oxygen-induced performance improvement, rather than on their underlying microbial mechanisms,^15,16^ resulting in few samples that were suitable for this study. Four samples for metagenomic sequencing were collected from a Napier grass-fed digester under anaerobic and ORP-controlled oxygenated conditions in duplicate runs, enabling the recovery of a complete genome of a highly abundant nanaerobe *Proteiniphilum* sp. from these systems.^17,18^ To study the abundance of nanaerobic bacteria and to minimize the operational impact of intrusive oxygen common in lab-scale systems, we also included metagenomic datasets from six mesophilic full-scale digesters.^32−34^ The samples were collected from different plant locations (Denmark, Spain, and China) and feedstock types (sewage sludge, animal manure, food waste, and maize silage). We lacked multi-omic datasets derived from the mesophilic digester fed with a lignocellulosic substrate for direct comparison with rumen systems. Therefore, we chose three datasets from thermophilic digesters fed livestock wastes as supplementary data.^35,36^ Finally, a metagenomic dataset from a deep subsurface oil reservoir (methanogenic system) served as a strict anaerobic control.^37^ In summary, data from 25 published samples were selected to elucidate the concept of nanaerobic digestion. The features of each ecosystem are summarized in Table 1, and the sequence characteristics of each dataset are given in Table S2.

**Table 1:**
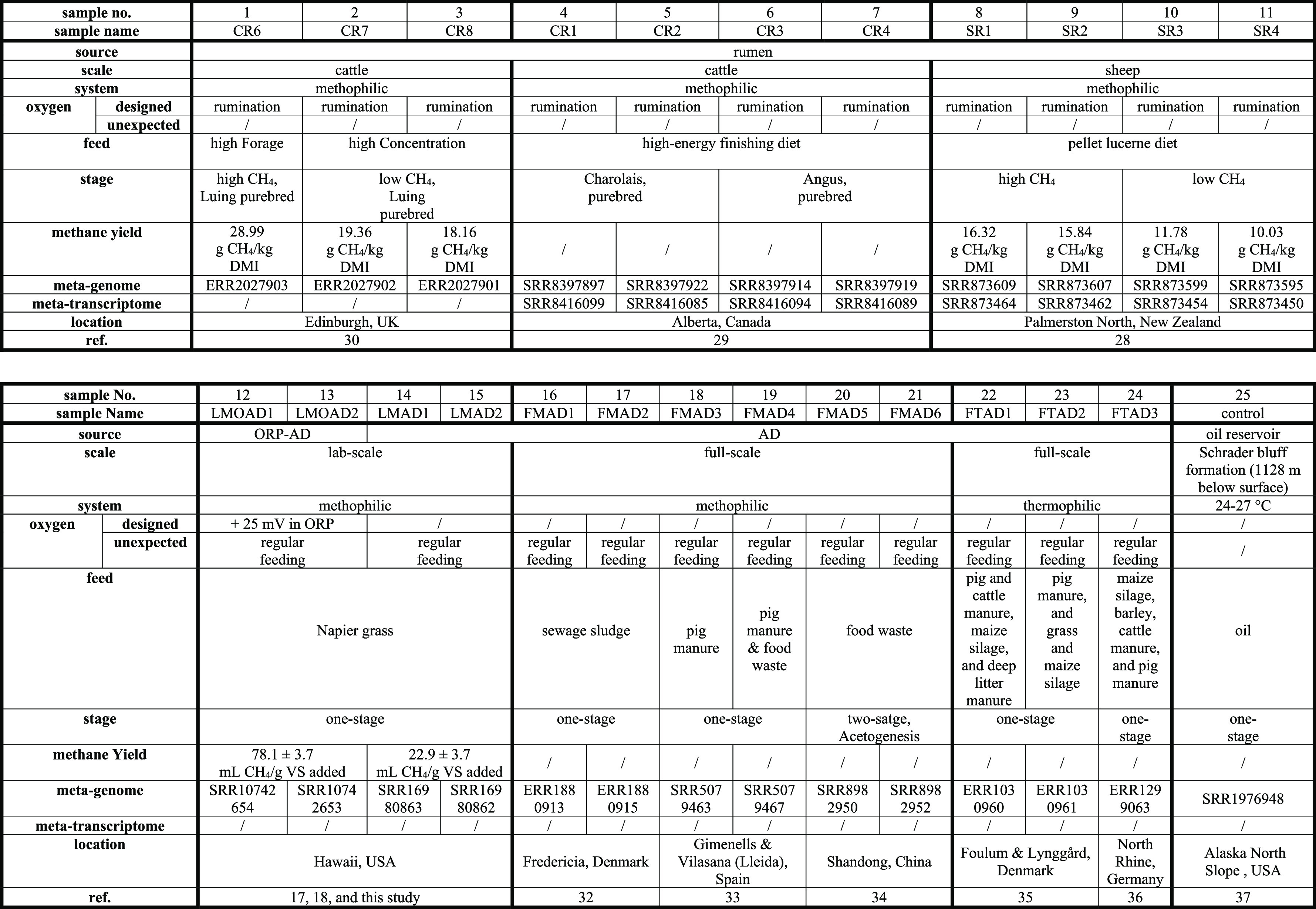
Characteristics of the Animal Rumina and Engineered Digesters^a^

a AD, anaerobic digester; ORP-AD, ORP-controlled oxygenated anaerobic digester.

### 2.2 Metagenomic and Metatranscriptomic Data Processing

Each metagenome was assembled and annotated to create its own reference gene database. Briefly, the raw paired-end metagenome reads were first filtered using Trimmomatic (version 0.36).^38^ The trimmed clean paired-end reads were then de novo-assembled into contigs using metaSPAdes from SPAdes (version 3.9.0).^39,40^ Due to the differences in sequence length, parameters for read trimming and assembly differed between samples (Table S2). For gene prediction and function annotation, the assembled contigs were then carried out by Prodigal program (version 3.0)^41^-incorporated Prokka Software (version 1.13) in metagenome mode.^42^ The process automatically found open reading frames (ORFs) and RNA regions, translated them into protein sequences, and searched them against a set of public databases (UniProt, Pfam, TIGRFAMs, and NCBI’s RefSeq) using BLAST and HMMER. Sequences shorter than 180 nucleotides were excluded, and an *e*-value threshold of 10^–6^ was used. For each dataset, the search result was combined to associate query genes with functional categories, including gene symbols, Enzyme Commission (EC) numbers, Clusters of Orthologous Groups (COG) terms, and protein products, creating a gene database for further functional quantification (the database of cytochrome *bd* oxidase genes for each sample is shown in Table S3).

To identify the functional profile, the metagenome and metatranscriptome reads were individually mapped back to the annotated assembly using Bowtie2 (version 2.2.9).^43^ First, an index database from assembly contigs was created using Bowtie2-build. Trimmed paired-end sequences were then mapped to the database with Bowtie2 using default parameters (local alignment, -D 20 -R 3 -N 0 -L 20 -i S,1,0.50 options). Finally, the number of reads mapped to the sequences in the assembly contigs was filtered and counted using Samtools (version 1.2)^44^ (the resulting numbers of each cytochrome *bd* oxidase gene for each sample are shown in Table S3). To facilitate the comparison between samples, mapped gene counts were normalized by gene length and the mean counts of housekeeping genes (values and equations for each sample are shown in Table S3). Four universal single-copy housekeeping genes (the RNA polymerase genes *rpoA*, *rpoB*, and *rpoC* and the recombinase gene *recA*) were used as the housekeeping genes in the procedure.^45,46^

To assign the taxonomic levels of cytochrome *bd* oxidase subunit genes (*cydA*, *appC*, and *ythA*) in the rumina, the sequences were extracted from the annotated metagenomic assembly of all the samples using Samtools (version 1.2)^44^ (sequence names from each sample are listed in Table S3). Then, sequences were analyzed with the following steps: (1) reads shorter than 300 bp were discarded, and chimeras were checked and removed with UCHIME (version 6.0);^47,48^ (2) sequences were translated and corrected with the *cydA* reference sequence using RDP FrameBot;^49^ (3) amino acid sequences were aligned with HMMER3.^50^ To construct a phylogenetic tree, reference sequences were obtained by searching against the NCBI nr database using the representative sequences. Then, a tree was built using a neighbor-joining method.^51^ To identify the microbial community composition, the program SortMeRNA (version 4.2)^52^ was used to extract 16S rRNA gene reads from the metagenomic datasets. Then, the extracted reads were taxonomically classified using the RDP classifier (version 2.2).^53^

### 2.3 Thermodynamic Calculations

The Gibbs free energy (Δ*G*) values were calculated in three steps according to Dolfing.^54^ First, the Gibbs free energy change for standard conditions (Δ*G*^0^) was calculated using the equation Δ*G*^0^ = ∑ *G*_f products_^0^ – ∑ *G*_f reactants_^0^, where *G*_f_^0^ is the Gibbs free energy of formation of a compound under standard conditions with a temperature of 25 °C, solutes at concentrations of 1 M, and gas partial pressure of 1 atmosphere. Next, a temperature correction was applied because most anaerobic digestion systems are operated under mesophilic conditions (∼35 °C) using the Gibbs–Helmholtz equation, , where *T*_ref_ is 298.15 K (25 °C), *T*_act_ is 308.15 K (35 °C), and Δ*H*_*T*ref_^0^ is the enthalpy of a chemical reaction under standard conditions by calculating the differences between total reactant and total product molar enthalpies. Finally, corrections for the actual solute concentrations and gas partial pressures were applied for specific conditions using the equation , where *R* is the universal gas constant (8.314 J/K mol), *A* and *B* represent the reactants, and *C* and *D* represent the products with *a*, *b*, *c*, and *d* representing the corresponding mole numbers in the reaction. For the calculation of thermodynamic constraints, Δ*G* was set to zero, and then the threshold conditions were calculated. Detailed procedures and assumptions are shown in Tables S4 and S5.

### 2.4 Statistical Analysis

The correlations between datasets were calculated using Pearson correlation in Excel. The comparison between groups was conducted using *t*-test. Differences were considered statistically significant when *p* < 0.05.

### 2.5 Data Availability

The metagenome sequencing reads from two lab-scale anaerobic digesters were submitted to the NCBI’s Sequence Read Archive under accession numbers SAMN23297125 to SAMN23297126. The cytochrome *bd* oxidase-related gene sequences retrieved from each metagenome were deposited at the FigShare Online Database (dx.doi.org/10.6084/m9.figshare.22776413).

## 3 Results and Discussion

### 3.1 Evaluation of Nanaerobic Respiration in Methanogenic Systems

**Figure 1:**
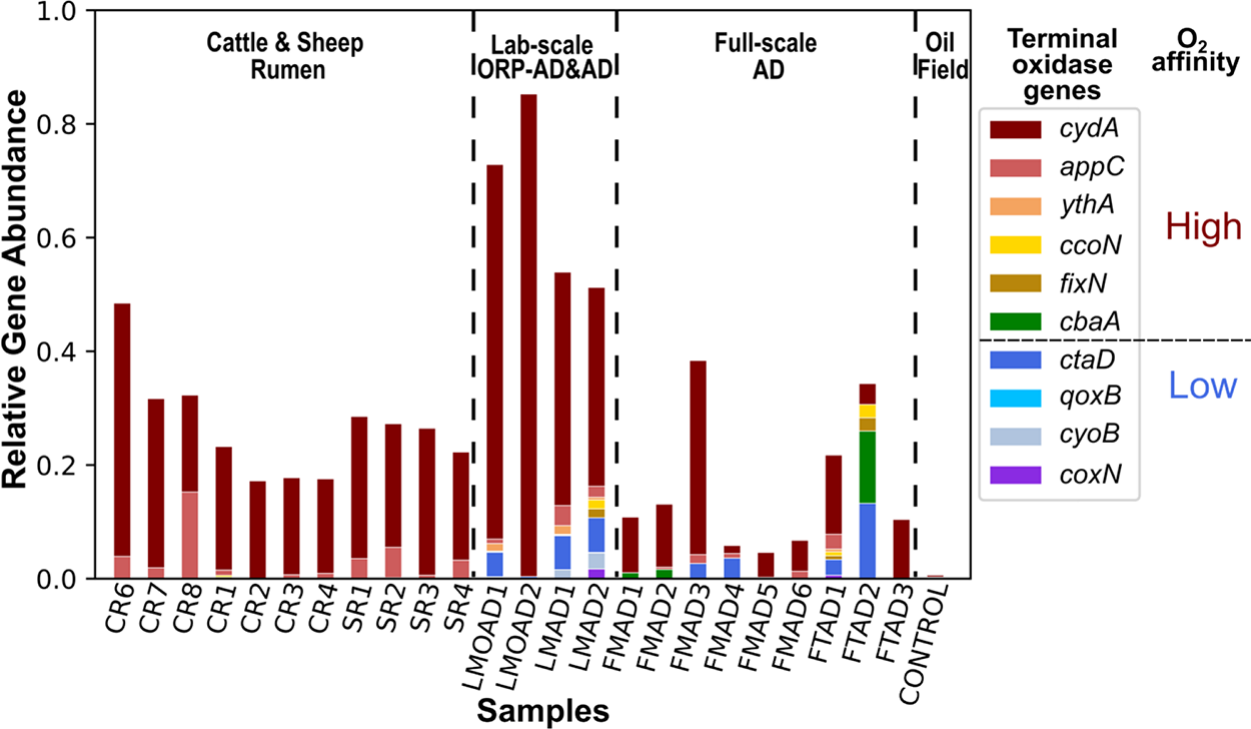
Distribution of terminal oxidase genes in animal rumina and engineered digesters. The sample names in the *x*-axis refer to the samples described in Table 1 and Table S2. The relative gene abundance was a ratio of normalized gene number between the genes of interest (see legend) and housekeeping genes (the RNA polymerase genes *rpoA*, *rpoB*, and *rpoC* and the recombinase gene *recA*) for each metagenome. The genes encoding the catalytic subunits of seven different terminal oxidases are shown. Among them, cytochrome *bb*_3_ (*coxN*), cytochrome *bo*_3_ (*cyoB*), cytochrome *aa*_3_-600 (*qoxB*), and cytochrome *aa*_3_ (*ctaD*) belong to the low-oxygen-affinity oxidases, whereas cytochrome *ba3* type (*cbaA*), cytochrome *cbb3* type (*fixN* and *ccoN*), and cytochrome *bd* type (type I, *cydA*; type II, *appC*; putative type, *ythA*) belong to the high-oxygen-affinity oxidases (details are given in Table S1).

To examine the possibility of aerobic respiration in methanogenic systems, we assessed the distribution of seven different terminal oxidases, including four low-oxygen-affinity variants [i.e., cytochrome *bb*_3_ (*coxN*), cytochrome *aa*_3_ (*ctaD*), cytochrome *aa*_3_-600 (*qoxB*), and cytochrome *bo*_3_ (*cyoB*)] and three high-oxygen-affinity variants [i.e., cytochrome *ba3* type (*cbaA*), cytochrome *cbb3* type (*fixN* and *ccoN*), and cytochrome *bd* type (type I (*cydA*), type II (*appC*), and putative type (*ythA*))] (Table S1). Figure 1 shows the normalized oxidase gene abundance for 11 rumen and 13 digester samples, as well as one sample from a deep subsurface oil reservoir (Table 1 and Table S3). Among all the samples, the oil reservoir microbiome had the lowest abundance of terminal oxidases and was used as a control for strict anaerobic conditions. The anaerobic digester samples maintained a higher abundance of terminal oxidases than the deep surface oil reservoir (*p* < 0.05), indicating that they may have regularly been exposed to oxygen. The abundance of terminal oxidases was greater in the lab-scale digesters than in full-scale digesters (*p* < 0.05), which is consistent with common difficulties of eliminating oxygen during lab-scale reactor operation. Findings from a previous lab-scale study of ORP-controlled oxygenated digesters provided insights into the impact of ultra-low oxygen levels on microbial populations.^17,18^ The overall abundances of terminal oxidase genes for ORP-controlled oxygenated conditions were significantly higher than for the corresponding conventional anaerobic conditions (*p* < 0.05), indicating that oxygen at nanomolar concentrations could induce terminal oxidase changes. Thus, the abundance of terminal oxidase genes may be a sensitive indicator of the response to environmental oxygen gradients. Based on these observations, a new understanding of ruminant digestion may be proposed. Animal rumina have generally been considered to be anaerobic environments as oxygen is typically undetectable (<250 nM) within 30 s after feeding.^55^ However, the rumen samples studied herein exhibited relatively high abundances of terminal oxidases, which were even higher than in several full-scale conventional anaerobic digesters (*p* < 0.05). These results imply that rumina commonly experience greater exposure to oxygen than expected. Rumination in animals, a process of rechewing the previously ingested rumen contents, might deliver oxygen for prolonged time periods, as this refeeding process occupies almost one-third of the lifetime of a healthy animal.^56^ By linking the different types of terminal oxidases with their respective oxygen affinity, more clues on ecosystem characteristics may be revealed. Herein, the high-oxygen-affinity variant cytochrome *bd* type (*cydA*, *appC*, and *ythA*) was dominant in all samples. As the nanaerobic respiration induced by cytochrome *bd* oxidase had been demonstrated to occur only at nanomolar oxygen levels,^12,13^ methanogenesis was likely maintained under nanaerobic rather than strict anaerobic conditions. However, samples from engineered systems usually contained low-oxygen-affinity oxidases (*ctaD*, *cbaA*, *etc.*), indicating apparent oxygen intrusions, which may happen during digester feeding.^57^ In contrast, rumen samples only contained the highest-oxygen-affinity cytochrome *bd* oxidase, suggesting a more reproducible and subtle fluctuation in oxygen levels in animal rumina. Therefore, the animal rumen system may represent an ideal model for nanaerobic digestion, where nanaerobes thrive in methanogenic environments, neither influenced by traditional facultative bacteria nor having an impact on anaerobic methanogens.

**Figure 2:**
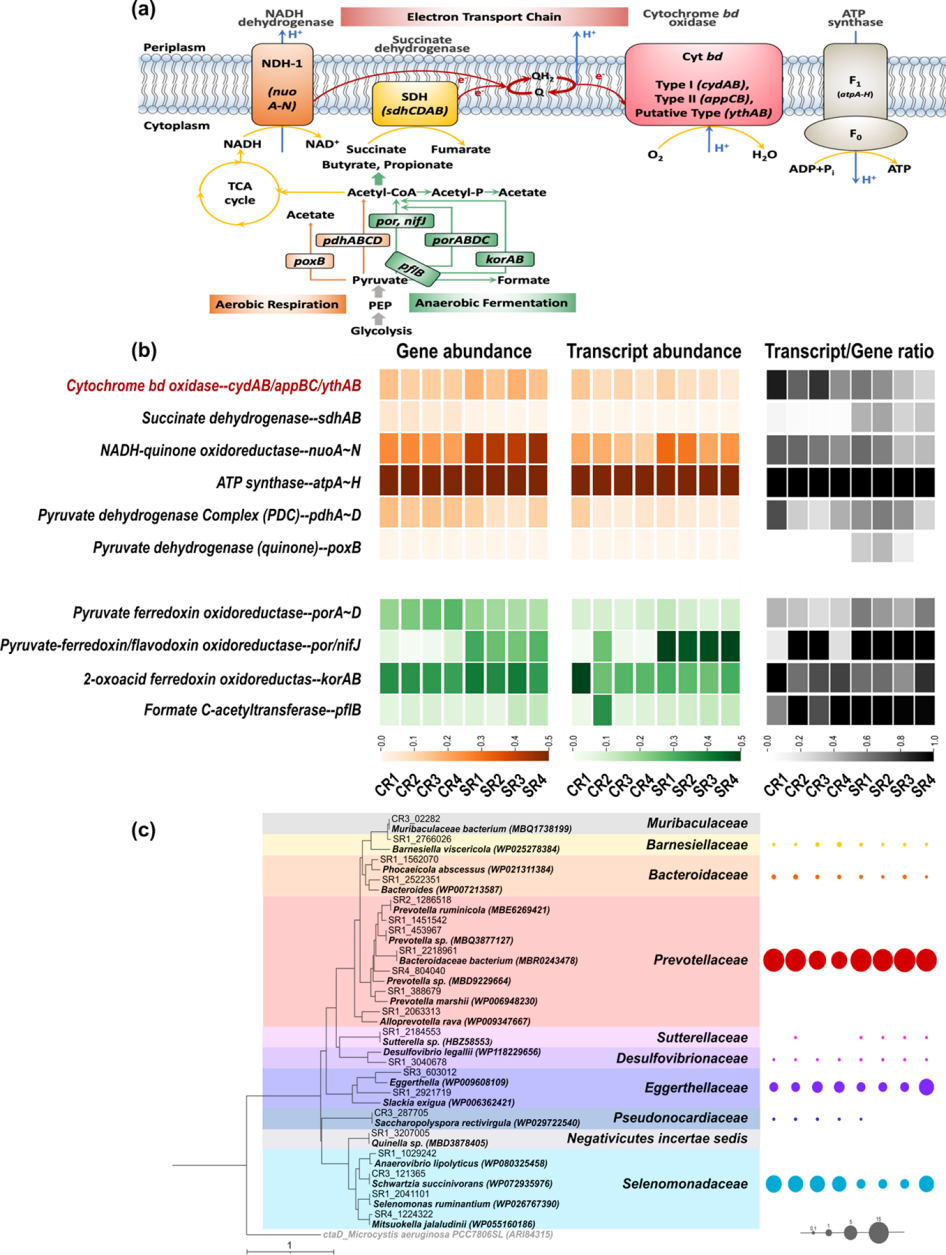
Metabolisms of nanaerobic respiration and anaerobic fermentation in four cattle and four sheep rumina. (a) Schematic diagram of key genes involved in the pyruvate conversion into acetyl-CoA and electron transfer chain (ETC). The nanaerobic respiration pathway is shown in orange, and the anaerobic fermentation pathway is marked in green. *poxB*, pyruvate dehydrogenase; *pdhABCD*, pyruvate dehydrogenase; *por*/*nifJ*, pyruvate-ferredoxin/flavodoxin oxidoreductase; *porABCD*, pyruvate ferredoxin oxidoreductase; *korAB*, 2-oxoacid ferredoxin oxidoreductase; *pflB*, formate *C*-acetyltransferase; *nuoA-N*, NADH-quinone oxidoreductase; *sdhAB*, succinate dehydrogenase; *cydAB/appBC/ythAB*, cytochrome *bd*-I/II/putative type oxidase. (b) Heatmap of normalized abundance of the above genes and transcripts in cattle and sheep rumina from metagenomic and metatranscriptomic analyses (for values, see Table S3). Genes and transcripts involved in the nanaerobic respiration pathway are shown in orange, genes and transcripts involved in the anaerobic fermentation pathway are shown in green, and transcript/gene ratios for both pathways are shown in gray. (c) Phylogenetic tree of cytochrome *bd* oxidase amino acid sequences (CydA, AppC, and YthA) retrieved from eight animal rumina and relative abundances of their affiliated bacterial families based on the extracted 16S rRNA gene sequences of each metagenome. The tree was constructed using 21 amino acid sequences, which were selected from a total of 598 sequences from the metagenome datasets. The phylogenetic tree constructed using 598 amino acid sequences retrieved from all the samples is shown in Figure S1. The sequence names for each sample are listed in Table S3.

As animal rumina harbor microbiomes that are consistent with the proposed concept of nanaerobic digestion, we further focused on ruminal microorganisms. The rumen microbial genes and their corresponding transcripts involved in aerobic respiration and anaerobic fermentation were analyzed to evaluate their co-occurrence. Since metatranscriptomic datasets were not available for some samples, eight rumen samples were examined. The functional profile (Figure 2a,b and Table S3) shows that all the cattle and sheep rumina possess and express the complete set of genes for aerobic respiration, including pyruvate oxidation, the tricarboxylic acid (TCA) cycle, and the electron transport chain. They further possess and express the genes associated with the anaerobic conversion of pyruvate into acetyl-CoA, which links to various fermentative metabolisms. These observations are in accordance with the end-fermentation products (e.g., acetate, propionate, and butyrate) produced in these cattle^58^ and sheep^59^ rumina. With respect to the ratios between transcripts and genes, gene expressions of pyruvate transformation under anaerobic conditions were generally higher than under aerobic conditions, in line with the final production of methane during rumen digestion. These results provide evidence that nanaerobic and anaerobic processes co-occur in rumen environments, which may contribute to the effectiveness of rumen digestion.

To evaluate the importance of nanaerobic respiration by ruminal nanaerobes, we also determined their relative abundances. We determined that most ruminal cytochrome *bd-*related gene sequences were affiliated with the family *Prevotellaceae* (phylum *Bacteroidetes*) (Figure 2c and Figure S1). Members of the family *Prevotellaceae* are common and dominant bacteria in rumina.^20,21^ They usually function as hydrolyzers and fermenters to degrade polysaccharides and peptides into a wide range of VFAs.^60^ Our data suggest that *Prevotellaceae* bacteria also live nanaerobic lifestyles in rumen environments: similar to facultative anaerobes, they can grow aerobically, yet similar to obligate anaerobes, they are only viable below DO levels of 2 μM.^12^ Moreover, nanaerobes belonging to the families *Selenomonadaceae* (phylum *Firmicutes*) and *Eggerthellaceae* (phylum *Actinobacteria*) were also found to be enriched in these rumen environments (Figure 2c and Figure S1). While the *in situ* detection of oxygen consumption by ruminal microorganisms has long been attributed to a transient action by ordinary facultative anaerobes,^55^ our results suggest that nanaerobic respiration is performed by these abundant rumen nanaerobes and appears to be an overlooked yet fundamental metabolism co-occurring with rumen methane production. The pattern of higher abundance of nanaerobes was also observed in our previous ORP-controlled oxygenated digesters as compared with their respective anaerobic control, in which the nanaerobe *Proteiniphilum* (phylum *Bacteroidetes*), which has genes for acetate fermentation and aerobic respiration (cytochrome *bd* as the sole oxidase), was always the dominant population (32–65%) (Figure S1).^17,18^ Other micro-oxygenated digesters were usually operated at a higher DO level of 0.1–1.0 mg/L (∼3–30 μM),^16^ which likely induce the presence of facultative anaerobes relying on conventional heme-copper oxidases to scavenge oxygen.^15,61^ However, since nanaerobes and obligate anaerobes (e.g., some methanogens) share similar niche preferences, nanaerobic operation may provide a promising development for methane production in engineered systems.

### 3.2 Characterization of the Synergetic Role of Hydrogenotrophic Methanogenesis with Nanaerobic Respiration

**Figure 3:**
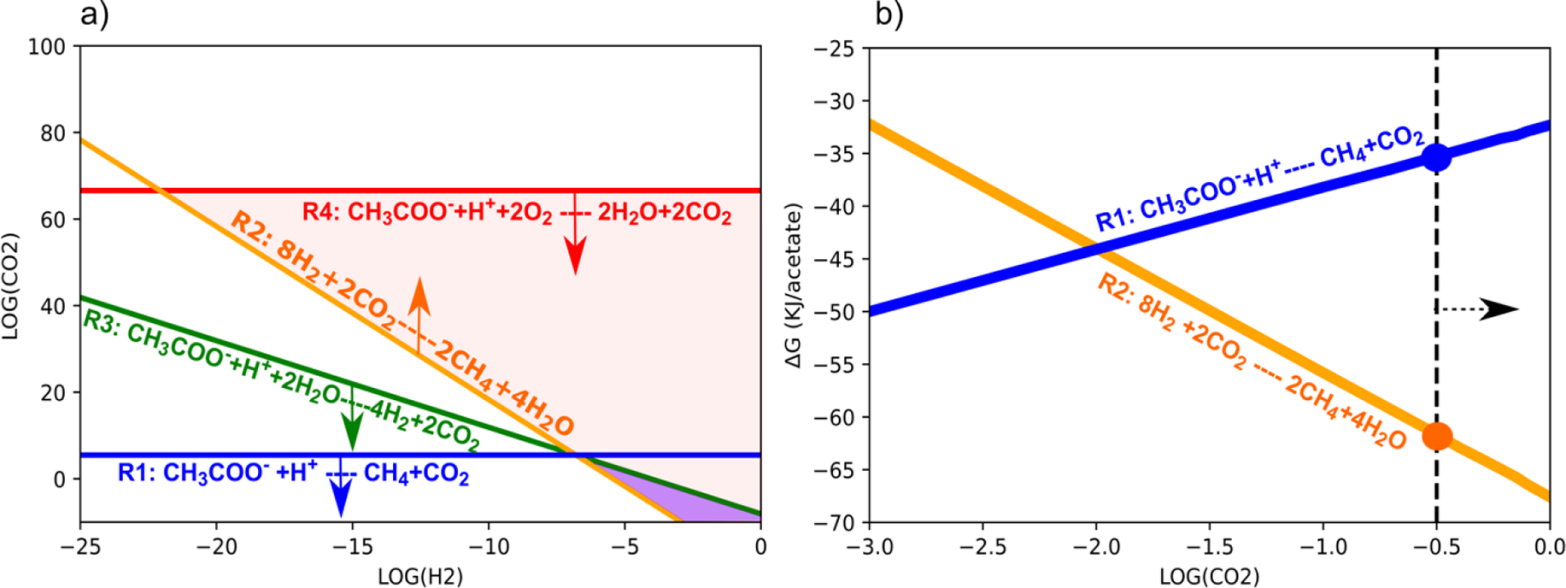
Thermodynamic constraints for methanogenesis. The calculations were performed for the following conditions: CH_4_ at 0.5 atm; O_2_ at 10^–8^ atm; acetate at 20 mM, pH = 6.5, 35 °C, and 1 atm, which is between the animal rumina and ORP-controlled oxygenated digesters mentioned in Table S4. (a) Partial pressures of H_2_ and CO_2_ are plotted as thermodynamic constraints for the conversion of acetate to methane. The lines represent the threshold at which the free energy change for each process is equal to zero. The direction of the arrows indicates conditions under which the processes become increasingly exergonic. Acetoclastic methanogenesis (R1) is shown in blue. Hydrogenotrophic methanogenesis (R2) is shown in orange. Syntrophic acetate oxidation (R3) is shown in green. Complete acetate oxidation (R4) is shown in red. The areas shaded in red and purple colors represent the coupling between complete acetate oxidation and hydrogenotrophic methanogenesis, whereas the area shaded in purple alone represents the linkage between syntrophic acetate oxidation and hydrogenotrophic methanogenesis. (b) CO_2_ partial pressure effect on the change in Gibbs free energy for methanogenesis. The dashed line between blue and orange dots represents the energy differences between acetoclastic and hydrogenotrophic methanogenesis at a specific CO_2_ partial pressure. Here, the dashed line represents a CO_2_ partial pressure of 0.3 atm. The direction of the arrow indicates the increase in CO_2_ partial pressure induced by nanaerobic respiration.

To understand nanaerobic ecosystems, it is important to characterize methanogenic populations. We found that rumina and the ORP-controlled oxygenated digesters share similarities in methanogenesis. In rumen environments, hydrogenotrophic methanogenesis is the dominant pathway for methane production,^62^ although acetate available at micromolar concentrations is sufficient to support acetoclastic methanogenesis.^63^ Similarly, hydrogenotrophic methanogenesis was observed to be prevalent in the ORP-controlled oxygenated digesters.^17,18^ To underpin the driving force behind these observations, we used thermodynamic calculations to evaluate the most likely pathway of methanogenesis under extremely low DO conditions. Herein, three possible routes of acetate conversion to methane were thermodynamically evaluated (Table 2 and Figures S4 and S5):^17,63^ (1) acetoclastic methanogenesis, (2) syntrophic acetate oxidation coupled with methanogenic CO_2_ reduction, and (3) complete acetate oxidation via nanaerobic respiration linked to CO_2_ reduction through hydrogenotrophic methanogenesis. First, the Gibbs free energy (Δ*G*) calculations show that hydrogenotrophic methanogenesis (R2) is more exergonic than acetoclastic methanogenesis (R1), indicating a thermodynamic advantage of methane production through CO_2_ reduction. Second, if acetoclastic methanogenesis is interrupted, hydrogenotrophic methanogenesis (R2) may couple with syntrophic acetate oxidation (R3), which usually relies on low H_2_ partial pressures.^64^ The thermodynamic constraints (Figure 3a), however, show that the reaction window for syntrophic methanogenesis (R3 + R2) is relatively small; the H_2_ partial pressure should fall between ∼10^–4^ and 10^–5^ atm for the conditions shown in Figure 3a. For the specific environmental conditions, syntrophic acetate oxidation (R3) is exergonic, but Δ*G* is close to zero in ORP-controlled oxygenated digesters, whereas the reaction is endergonic in rumina (Table 2 and Tables S4 and S5). These data are consistent with the low relative abundance of syntrophic bacteria (less than 1%) in the ORP-controlled oxygenated digester.^17^ In rumina, due to the relatively high H_2_ partial pressures (between 2 × 10^–4^ and 1 × 10^–2^ atm), reductive acetogens, bacteria that reversely synthesize acetate from CO_2_ and H_2_, are found frequently.^65^ Thus, there might be alternative pathways to support hydrogenotrophic methanogenesis. Finally, if DO is present at nanomolar levels, nanaerobes may enable complete acetate degradation into CO_2_ (R4) using the cytochrome *bd* oxidase, thereby providing a new route to link CO_2_ reduction (R2) by hydrogenotrophic methanogenesis. Thermodynamic calculations (Figure 3a) verify that a substantial window of opportunity exists for this route, possibly bypassing the thermodynamically limited reaction of syntrophic acetate oxidation. Meanwhile, Δ*G* calculations show that such nanaerobic methanogenesis (R4 + R2) provides higher energy for ATP yield and biomass synthesis, which may benefit the proliferation of nanaerobes, and in turn may improve their fermentation capabilities. Our previous observation in ORP-controlled oxygenated digestion indeed confirmed that the nanaerobe *Proteiniphilum* became dominant and therefore alleviated VFA stress and increased overall digestibility.^17,18^ The abundance of nanaerobic *Prevotellaceae* in rumina as observed in this study also suggest their collaboration with hydrogenotrophic methanogens. As additional conversion of VFAs leads to an increase in CO_2_ partial pressure, hydrogenotrophic methanogenesis (R2) becomes more exergonic than acetoclastic methanogenesis (R1) (Figure 3b). Therefore, hydrogenotrophic methanogenesis coupled with nanaerobic respiration may constitute the main pathway for methane generation in oxygenated methanogenic systems.

**Table 2:** Gibbs Free Energies of Three Pathways for the Conversion of Acetate to Methane in Animal Rumina and ORP-Controlled Oxygenated Anaerobic Digesters^a^

						Δ*G*_308.15K,1atm_ (kJ/reaction)		
pathway no.	pathway	reaction no.	reaction	Δ*G*_298.15K_ (kJ/reaction)	Δ*G*_308.15K_ (kJ/reaction)	rumen (high roughage)	rumen (high concentrate)	ORP-AD (replicate 1–day 149)
I	acetoclastic methanogenesis	1	CH_3_COO^–^ + H^+^ → CH_4_ + CO_2_	–75.8	–78.9	–37.6	–44.4	–29.3
								
II	syntrophic acetate oxidation	3	CH_3_COO^–^ + H^+^+2H_2_O → 4H_2_ + 2CO_2_	54.9	47.6	24.9	24.1	–4.6
	hydrogenotrophic methanogenesis	2	8H_2_ + 2CO_2_ → 2CH_4_ + 4H_2_O	–261.6	–252.8	–124.7	–136.7	–49.0
	sum	3 + 2	CH_3_COO^–^ + H^+^+4H_2_ → 2CH_4_ + 2H_2_O	–206.7	–205.2	–99.8	–112.5	–53.6
								
III	complete acetate oxidation	4	CH_3_COO^–^ + H^+^+2O_2_ → 2H_2_O + 2CO_2_	–893.7	–894.4	–784.7	–790.5	–737.6
	hydrogenotrophic methanogenesis	2	8H_2_ + 2CO_2_ → 2CH_4_ + 4H_2_O	–261.6	–252.8	–124.7	–136.7	–49.0
	sum	4 + 2	CH_3_COO^–^ + H^+^+8H_2_ + 2O_2_ → 2CH_4_ + 6H_2_O	–1155.3	–1147.2	–909.4	–927.1	–786.6

a High roughage, animal fed with high-roughage diet; high concentrate, animal fed with high-concentrate diet; ORP-AD, ORP-controlled oxygenated anaerobic digester; replicate 1–day 149, condition at the day 149 of the first operation period.

### 3.3 Evaluation of Methane Production in Nanaerobic Digestion

With the synergistic interaction between nanaerobic respiration and hydrogenotrophic methanogenesis, the overall digestion performances can be improved in both animal rumina and oxygenated digesters (Table 3). While typical nanaerobic digesters and normal oxygenated digesters may have different microbiomes, they share similar performance trends. We thus discuss the oxygenated digesters as well as the ORP-controlled oxygenated digesters to link their phenotypes with animal rumen phenotypes. The enhancement of methane yield has been identified as the main benefit in oxygenated digesters.^15,16^ Specifically, in our ORP-controlled oxygenated digestion of Napier grass, a typical lignocellulosic biomass, a 3.4-fold increase in methane yield, a 2.3-fold improvement in VS removal, and a 2.1-fold reduction in VFA concentration were achieved relative to an unstable strict anaerobic digester, which was on the verge of failure.^17^ Such improvement was found to be closely associated with the cytochrome *bd*-encoding nanaerobe *Proteiniphilum.*(18) Similarly, ruminants are well known for their ability to efficiently degrade fibers and the high emission of methane gas.^19^ Although *in situ* oxygen consumption by rumen microbiota has been previously reported,^55^ the rumen is commonly regarded an anaerobic environment.^66^ The importance of introducing nanomolar levels of oxygen during regurgitation and reswallowing (i.e., rumination) has been mostly overlooked, possibly due to the relatively high detection limits of oxygen electrodes used in rumen environments (>250 nM).^27^ Interestingly, in this study, when mapping four sheep rumina methane yield data (Table 1) to their corresponding oxygen-related gene profiles (Figure 2b and Table S3), we found that methane yield correlated with the abundance (*r* = 0.884, *p* < 0.05) and expression (*r* = 0.996, *p* < 0.05) of cytochrome *bd* oxidase (*cydA*, *appC*, and *ythA*) and even more strongly with the ratio of transcript over gene abundance (*r* = 0.999, *p* < 0.05). The correlation was also found between the methane yield and cytochrome *bd* oxidase gene abundance in three cattle rumina (*r* = 0.991, *p* < 0.05, Table 1 and Table S3), suggesting methane enhancement by oxygen occurrence in animal rumina. This phenomenon can be further confirmed through current practices in ruminant dietary management. Using high-concentrate diets (grain) instead of high-roughage diets (forage) has been an effective strategy for methane emission reduction.^67^ If the degree of oxygenation is linked to the frequency of rumination induced by different diets, a new connection may be established. A high-roughage diet requires more chewing and rumination,^68^ leading to more frequent introduction of oxygen, which results in a higher methane emission. In contrast, the high-concentrate diet with readily digestible carbohydrates requires less rumination^68^ and therefore utilizes less oxygen and produces less methane. On this basis, ultra-low oxygenation during rumination may be a key factor to regulate ruminant methane emission.

**Table 3:** Four Common Phenotypes Shared by Animal Rumina and Micro-Oxygenated Anaerobic Digesters^a^

no.	characteristics	ruminants with roughage (high O_2_) and concentrate (low O_2_) diets	ref	micro-oxygenated AD process	ref
1	Methane yield correlated with cytochrome *bd* oxidase gene.	Methane yields show a positive correlation with the abundance of cytochrome *bd*-encoded genes in four sheep samples.	this study	Methane enhancement was accompanied by an increase in the abundance of cytochrome *bd*-encoding nanaerobe *Proteiniphilum*.	(17), (18)
					
2	Methane yield correlated with oxygen concentration.	High-roughage diet leads to higher emission of methane, whereas high-concentrate diet produces less methane.	(67)	Microaeration improves organic degradation and enhances the methane yield in AD processes.	(15), (16)
					
3	VFA concentration and pH value	High-concentrate diet is frequently results in acute ruminal acidosis.	(69)	Microaeration controls VFA accumulation, alleviates pH drop, and stabilizes the AD process.	(17)
					
4	VFA type	High-roughage diet favors acetate production, whereas high-concentrate diet promotes propionate production.	(67)	Microaeration enhances acetate formation but decreases propionate accumulation, thereby increasing the ratio of acetate to propionate.	(70), (71)

a Micro-oxygenated AD, micro-oxygenated anaerobic digester.

Furthermore, VFA patterns are also influenced by the oxygenation levels in both rumina and oxygenated digesters. First, although the high-concentrate diet is beneficial for ruminant methane mitigation, it frequently results in acute acidosis.^69^ This may result from excess feed intake but insufficient rumination, which lowers oxygenation and therefore hinders VFA transformation through nanaerobic respiration. In the ORP-controlled oxygenated digesters, at an OLR (5 g VS/L/day), which is much higher than typical OLRs (1–4 g VS/L/day),^64^ small amounts of oxygen rapidly led to the consumption of accumulated VFAs without external alkalinity supplementation.^17^ Second, the types of VFAs (e.g., acetate, propionate, and butyrate) produced are dependent on the diet. Ruminants usually produce more acetate when fed a high-roughage diet, whereas they generate more propionate when fed with a concentrate-based diet.^67^ Similarly, several micro-oxygenated digestion studies also reported oxygen-enhanced acetate formation and decreased propionate accumulation, leading to a high ratio of acetate to propionate.^70,71^ Therefore, promoting ruminal oxygenation may be effective in balancing rumen health and methane production.

### 3.4 The Concept of Nanaerobic Digestion

**Figure 4:**
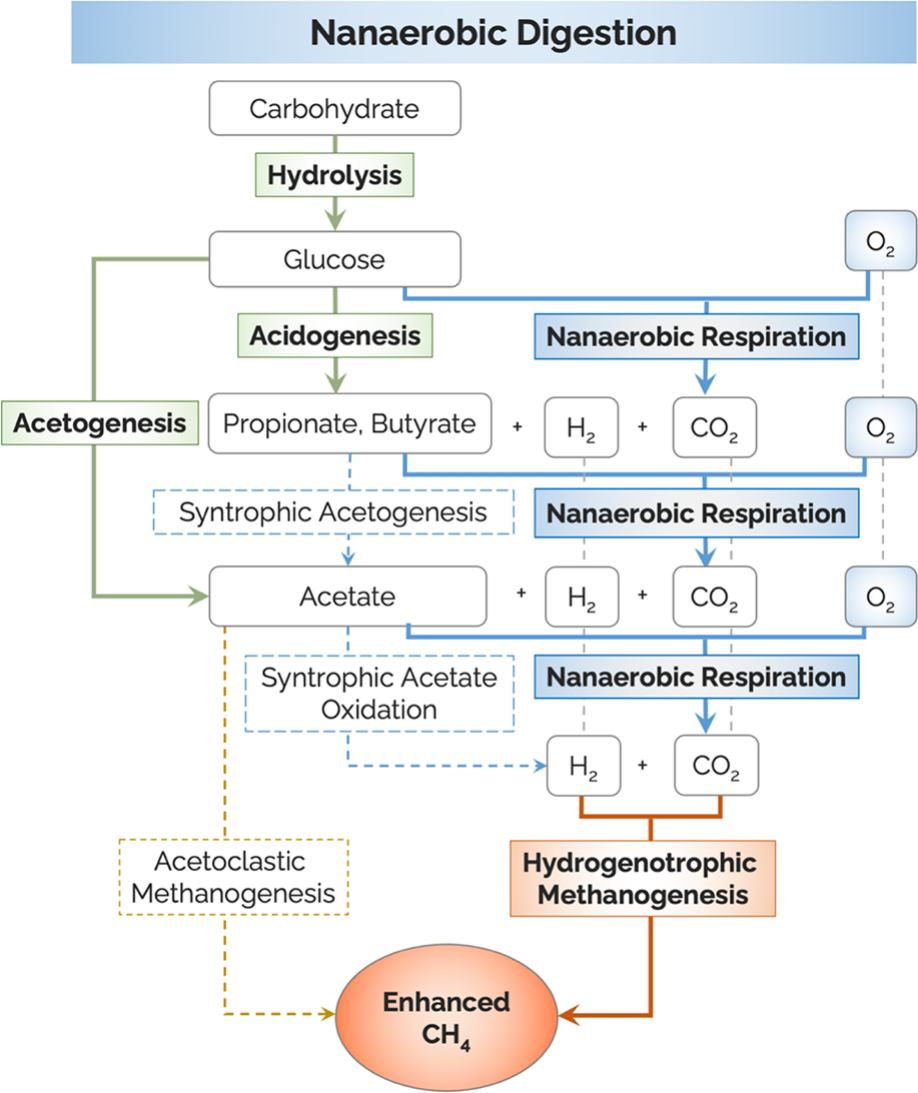
Schematic illustration of the main steps of nanaerobic digestion, revised from Nguyen et al.^17^ and Wu et al.^18^ Solid lines represent dominant pathways, whereas dashed lines represent minor pathways. First, complex organics are hydrolyzed and fermented into simple organic acids by three conventional steps (hydrolysis, acidogenesis, and acetogenesis). Then, due to the presence of nanomolar levels of DO, simple organics are completely oxidized to CO_2_ via nanaerobic respiration, which promotes methane production through hydrogenotrophic methanogenesis.

We demonstrated the occurrence of overlooked nanaerobes as well as their significance in animal rumina and ORP-controlled oxygenated digesters. Nanaerobic digestion consists of a five-step pathway, which includes nanaerobic respiration in addition to four traditional anaerobic digestion steps for efficient organic degradation and enhanced methane production (Figure 4). In the presence of nanomolar concentrations of DO, nanaerobes can co-exist with methanogens, and anaerobic fermentation and nanaerobic respiration are performed simultaneously. Like the classical acidogenic and acetogenic fermenters, nanaerobes can convert organic compounds into VFAs and hydrogen. Meanwhile, they also can aerobically degrade organic compounds and VFAs into CO_2_ using cytochrome *bd* oxidase. Such co-occurring processes not only bypass the thermodynamically unfavorable syntrophic VFA oxidation reaction but also accelerate and promote the degradation of organics, especially VFAs, and result in the enhanced production of methane by hydrogenotrophic methanogens.

### 3.5 Recommendations for Environmental Practice

Since nanomolar DO levels are ubiquitous, although often not detected, in both natural and engineered methanogenic systems, the concept of nanaerobic digestion may provide additional perspectives for understanding the global methane fluxes, such as methane emissions from ocean^72^ and wetland^73^ sediments, rumen digestion and ruminant productivity, and energy recovery from biomass.

The notion that rumina behave like nanaerobic systems rather than purely anaerobic systems advocates for a balance between animal productivity improvement and methane emission reduction. Methane emission may be an evolutionary advantage for ruminants. Rumen consortia containing nanaerobes may allow the animal to intake large amounts of fiber to achieve maximum growth without experiencing ruminal acidosis. Because of the presence of oxygen introduced during rumination, VFA compounds that are not promptly absorbed by the animal may be rapidly degraded into CO_2_ via nanaerobic respiration, playing a buffering role to maintain the VFA balance between animal assimilation and microbial fermentation. Meanwhile, we speculate that greater methane production, which results from the additional degradation of excess fiber and VFAs, may induce intense eructation and therefore physically stop further feed consumption, representing a feedback signal for balanced feeding of the animal. Therefore, both ruminant health and growth should be considered in combination when developing strategies for methane mitigation.

The proposed nanaerobic digestion brings together two fields of anaerobic digestion research, namely, rumen-inoculated^22−24^ and micro-oxygenated digesters.^15,16^ Given the evidence that rumen digestion has similarities to ORP-controlled oxygenated digestion, a nanaerobic environment may be needed for the maintenance of rumen microorganisms in a continuously operated bioreactor, avoiding the washout or inactivity of rumen bacteria from the system. Meanwhile, considering the niche sharing of nanaerobes and methanogens at nanomolar (or lower) DO concentrations, our findings may provide guidance on appropriate DO concentrations for the operation of micro-oxygenated anaerobic digesters. Overall, nanaerobic operation and nanaerobe enrichment may improve energy recovery for digestion applications.

## Funding

This work was supported by a grant from Imperial Seed grant (CIVE F14020) and partly by the National Institute of Food and Agriculture, U.S. Department of Agriculture, under award number 2013-67022-21177. S.S. was supported by an Integrated Training in Microbial Systems Fellowship funded by the Burroughs Welcome Fund, the University of Michigan Rackham Predoctoral Fellowship, and a Water Environment Federation Canham Graduate Studies Scholarship.

## Supporting Information Available

Sequence datasets (XLSX)
Gene or transcript abundance (XLSX)
thermodynamic calculations (XLSX)
Different bacterial terminal oxidases and thermodynamic conditions (PDF)
Phylogenetic tree (PDF)

## References

[ref1] Tollefson J. Scientists raise alarm over dangerously fast growth in atmospheric methane. Nature 2022, 10.1038/d41586-022-00312-2 35136235

[ref2] Sherwood J. The significance of biomass in a circular economy. Bioresour. Technol. 2020, 300, 122755 10.1016/j.biortech.2020.122755. 31956060

[ref3] Goldblatt C.; Lenton T. M.; Watson A. J. Bistability of atmospheric oxygen and the Great Oxidation. Nature 2006, 443, 683–686. 10.1038/nature05169. 17036001

[ref4] Fenchel T.; Finlay B. J. Ecology and evolution in anoxic worlds (Oxford Univ. Press, 1995).

[ref5] Devol A. H. Bacterial oxygen uptake kinetics as related to biological processes in oxygen deficient zones of the oceans. Deep-Sea Res. 1978, 25, 137–146. 10.1016/0146-6291(78)90001-2.

[ref6] Rutten M. G. The history of atmospheric oxygen. Space Life Sci. 1970, 2, 5–17. 10.1007/BF00928950. 5521892

[ref7] Crichton P. G.; Albury M. S.; Affourtit C.; Moore A. L. Mutagenesis of the *Sauromatum guttatum* alternative oxidase reveals features important for oxygen binding and catalysis. Biochim. Biophys. Acta, Bioenerg. 2010, 1797, 732–737. 10.1016/j.bbabio.2009.12.010. 20026041

[ref8] Rice C. W.; Hempfling W. P. Oxygen-limited continuous culture and respiratory energy conservation in *Escherichia coli*. J. Bacteriol. 1978, 134, 115–124. 10.1128/jb.134.1.115-124.1978. 25879 PMC222225

[ref9] D’mello R.; Hill S.; Poole R. K. The oxygen affinity of cytochrome *bo’* in *Escherichia coli* determined by the deoxygenation of oxyleghemoglobin and oxymyoglobin: Km values for oxygen are in the submicromolar range. J. Bacteriol. 1995, 177, 867–870. 10.1128/jb.177.3.867-870.1995. 7836332 PMC176676

[ref10] Jackson R. J.; Elvers K. T.; Lee L. J.; Gidley M. D.; Wainwright L. M.; Lightfoot J.; Park S. F.; Poole R. K. Oxygen reactivity of both respiratory oxidases in *Campylobacter jejuni*: the *cydAB* genes encode a cyanide-resistant, low-affinity oxidase that is not of the cytochrome *bd* type. J. Bacteriol. 2007, 189, 1604–1615. 10.1128/JB.00897-06. 17172349 PMC1855770

[ref11] D’mello R.; Hill S.; Poole R. K. The cytochrome *bd* quinol oxidase in *Escherichia coli* has an extremely high oxygen affinity and two oxygen-binding haems: implications for regulation of activity in vivo by oxygen inhibition. Microbiology 1996, 142, 755–763. 10.1099/00221287-142-4-755. 8936304

[ref12] Baughn A. D.; Malamy M. H. The strict anaerobe *Bacteroides fragilis* grows in and benefits from nanomolar concentrations of oxygen. Nature 2004, 427, 441–444. 10.1038/nature02285. 14749831

[ref13] Stolper D. A.; Revsbech N. P.; Canfield D. E. Aerobic growth at nanomolar oxygen concentrations. Proc. Natl. Acad. Sci. U. S. A. 2010, 107, 18755–18760. 10.1073/pnas.1013435107. 20974919 PMC2973883

[ref14] García-Bayona L.; Coyne M. J.; Hantman N.; Montero-Llopis P.; Von S. S.; Ito T.; Malamy M. H.; Basler M.; Barquera B.; Comstock L. E. Nanaerobic growth enables direct visualization of dynamic cellular processes in human gut symbionts. Proc. Natl. Acad. Sci. U. S. A. 2020, 117, 24484–24493. 10.1073/pnas.2009556117. 32938803 PMC7533675

[ref15] Nguyen D.; Khanal S. K. A little breath of fresh air into an anaerobic system: How microaeration facilitates anaerobic digestion process. Biotechnol. Adv. 2018, 36, 1971–1983. 10.1016/j.biotechadv.2018.08.007. 30144516

[ref16] Chen Q.; Wu W.; Qi D.; Ding Y.; Zhao Z. Review on microaeration-based anaerobic digestion: state of the art, challenges, and prospectives. Sci. Total Environ. 2020, 710, 136388 10.1016/j.scitotenv.2019.136388. 31923694

[ref17] Nguyen D.; Wu Z.; Shrestha S.; Lee P. H.; Raskin L.; Khanal S. K. Intermittent micro-aeration: New strategy to control volatile fatty acid accumulation in high organic loading anaerobic digestion. Water Res. 2019, 166, 115080 10.1016/j.watres.2019.115080. 31541792

[ref18] Wu Z.; Nguyen D.; Lam T. Y. C.; Zhuang H.; Shrestha S.; Raskin L.; Khanal S. K.; Lee P.-H. Synergistic association between cytochrome *bd*-encoded *Proteiniphilum* and reactive oxygen species (ROS)-scavenging methanogens in microaerobic-anaerobic digestion of lignocellulosic biomass. Water Res. 2021, 190, 116721 10.1016/j.watres.2020.116721. 33326896

[ref19] Chang J.; Peng S.; Ciais P.; Saunois M.; Dangal S. R.; Herrero M.; Havlík P.; Tian H.; Bousquet P. Revisiting enteric methane emissions from domestic ruminants and their δ^13^C_CH4_ source signature. Nat. Commun. 2019, 10, 3420 10.1038/s41467-019-11066-3. 31366915 PMC6668425

[ref20] Huws S. A.; Creevey C. J.; Oyama L. B.; Mizrahi I.; Denman S. E.; Popova M.; Muñoz-Tamayo R.; Forano E.; Waters S. M.; Hess M.; Tapio I.; Smidt H.; Krizsan S. J.; Yáñez-Ruiz D. R.; Belanche A.; Guan L.; Gruninger R. J.; McAllister T. A.; Newbold C. J.; Roehe R.; Dewhurst R. J.; Snelling T. J.; Watson M.; Suen G.; Hart E. H.; Kingston-Smith A. H.; Scollan N. D.; do Prado R. M.; Pilau E. J.; Mantovani H. C.; Attwood G. T.; Edwards J. E.; McEwan N. R.; Morrisson S.; Mayorga O. L.; Elliott C.; Morgavi D. P. Addressing global ruminant agricultural challenges through understanding the rumen microbiome: past, present, and future. Front. Microbiol. 2018, 9, 2161 10.3389/fmicb.2018.02161. 30319557 PMC6167468

[ref21] O’Hara E.; Neves A. L. A.; Song Y.; Guan L. L. The role of the gut microbiome in cattle production and health: driver or passenger?. Annu. Rev. Anim. Biosci. 2020, 8, 199–220. 10.1146/annurev-animal-021419-083952. 32069435

[ref22] Shrestha S.; Fonoll X.; Khanal S. K.; Raskin L. Biological strategies for enhanced hydrolysis of lignocellulosic biomass during anaerobic digestion: current status and future perspectives. Bioresour. Technol. 2017, 245, 1245–1257. 10.1016/j.biortech.2017.08.089. 28941664

[ref23] Fonoll X.; Shrestha S.; Khanal S. K.; Dosta J.; Mata-Alvarez J.; Raskin L. Understanding the Anaerobic Digestibility of Lignocellulosic Substrates Using Rumen Content as a Cosubstrate and an Inoculum. ACS ES&T Eng. 2021, 1, 424–435. 10.1021/acsestengg.0c00164.

[ref24] Fonoll X.; Zhu K.; Aley L.; Shrestha S.; Raskin L. Simulating Rumen Conditions using an Anaerobic Dynamic Membrane Bioreactor to Enhance Hydrolysis of Lignocellulosic Biomass. bioRxiv 2023, 2023-02 10.1101/2023.02.20.529314. 38184844

[ref25] Espey M. G. Role of oxygen gradients in shaping redox relationships between the human intestine and its microbiota. Free Radical Biol. Med. 2013, 55, 130–140. 10.1016/j.freeradbiomed.2012.10.554. 23127782

[ref26] Brune A. Symbiotic digestion of lignocellulose in termite guts. Nat. Rev. Microbiol. 2014, 12, 168–180. 10.1038/nrmicro3182. 24487819

[ref27] Scott R. I.; Yarlett N.; Hillman K.; Williams A. G.; Lloyd D.; Williams T. N. The presence of oxygen in rumen liquor and its effects on methanogenesis. J. Appl. Microboil. 1983, 55, 143–149. 10.1111/j.1365-2672.1983.tb02658.x.

[ref28] Allen M. S. Physical constraints on voluntary intake of forages by ruminants. J. Anim. Sci. 1996, 74, 3063–3075. 10.2527/1996.74123063x. 8994921

[ref29] Shi W.; Moon C. D.; Leahy S. C.; Kang D.; Froula J.; Kittelmann S.; Fan C.; Deutsch S.; Gagic D.; Seedorf H.; Kelly W. J.; Atua R.; Sang C.; Soni P.; Li D.; Pinares-Patiño C. S.; McEwan J. C.; Janssen P. H.; Chen F.; Visel A.; Wang Z.; Attwood G. T.; Rubin E. M. Methane yield phenotypes linked to differential gene expression in the sheep rumen microbiome. Genome Res. 2014, 24, 1517–1525. 10.1101/gr.168245.113. 24907284 PMC4158751

[ref30] Li F.; Hitch T. C. A.; Chen Y.; Creevey C. J.; Guan L. L. Comparative metagenomic and metatranscriptomic analyses reveal the breed effect on the rumen microbiome and its associations with feed efficiency in beef cattle. Microbiome 2019, 7, 1–21. 10.1186/s40168-019-0618-5. 30642389 PMC6332916

[ref31] Auffret M. D.; Dewhurst R. J.; Duthie C. A.; Rooke J. A.; John Wallace R.; Freeman T. C.; Stewart R.; Watson M.; Roehe R. The rumen microbiome as a reservoir of antimicrobial resistance and pathogenicity genes is directly affected by diet in beef cattle. Microbiome 2017, 5, 1–11. 10.1186/s40168-017-0378-z. 29228991 PMC5725880

[ref32] Kirkegaard R. H.; Dueholm M. S.; McIlroy S. J.; Nierychlo M.; Karst S. M.; Albertsen M.; Nielsen P. H. Genomic insights into members of the candidate phylum Hyd24-12 common in mesophilic anaerobic digesters. ISME J. 2016, 10, 2352–2364. 10.1038/ismej.2016.43. 27058503 PMC5030696

[ref33] Ruiz-Sánchez J.; Campanaro S.; Guivernau M.; Fernández B.; Prenafeta-Boldú F. X. Effect of ammonia on the active microbiome and metagenome from stable full-scale digesters. Bioresour. Technol. 2018, 250, 513–522. 10.1016/j.biortech.2017.11.068. 29197774

[ref34] Li K.; Wang K.; Wang J.; Yuan Q.; Shi C.; Wu J.; Zuo J. Performance assessment and metagenomic analysis of full-scale innovative two-stage anaerobic digestion biogas plant for food wastes treatment. J. Cleaner Prod. 2020, 264, 121646 10.1016/j.jclepro.2020.121646.

[ref35] Mosbæk F.; Kjeldal H.; Mulat D. G.; Albertsen M.; Ward A. J.; Feilberg A.; Nielsen J. L. Identification of syntrophic acetate-oxidizing bacteria in anaerobic digesters by combined protein-based stable isotope probing and metagenomics. ISME J. 2016, 10, 2405–2418. 10.1038/ismej.2016.39. 27128991 PMC5030692

[ref36] Maus I.; Koeck D. E.; Cibis K. G.; Hahnke S.; Kim Y. S.; Langer T.; Kreubel J.; Erhard M.; Bremges A.; Off S.; Stolze Y.; Jaenicke S.; Goesmann A.; Sczyrba A.; Scherer P.; König H.; Schwarz W. H.; Zverlov V. V.; Liebl W.; Püler A.; Schlüter A.; Klocke M. Unraveling the microbiome of a thermophilic biogas plant by metagenome and metatranscriptome analysis complemented by characterization of bacterial and archaeal isolates. Biotechnol. Biofuels 2016, 9, 1–28. 10.1186/s13068-016-0581-3. 27525040 PMC4982221

[ref37] Hu P.; Tom L.; Singh A.; Thomas B. C.; Baker B. J.; Piceno Y. M.; Andersen G. L.; Banfield J. F. Genome-resolved metagenomic analysis reveals roles for candidate phyla and other microbial community members in biogeochemical transformations in oil reservoirs. MBio 2016, 7, e01669–e01615. 10.1128/mBio.01669-15. 26787827 PMC4725000

[ref38] Bolger A. M.; Lohse M.; Usadel B. Trimmomatic: a flexible trimmer for Illumina sequence data. Bioinformatics 2014, 30, 2114–2120. 10.1093/bioinformatics/btu170. 24695404 PMC4103590

[ref39] Bankevich A.; Nurk S.; Antipov D.; Gurevich A. A.; Dvorkin M.; Kulikov A. S.; Lesin V. M.; Nikolenko S. I.; Pham S.; Prjibelski A. D.; Pyshkin A. V.; Sirotkin A. V.; Vyahhi N.; Tesler G.; Alekseyev M. A.; Pevzner P. A. SPAdes: a new genome assembly algorithm and its applications to single-cell sequencing. J. Comput. Biol. 2012, 19, 455–477. 10.1089/cmb.2012.0021. 22506599 PMC3342519

[ref40] Nurk S.; Meleshko D.; Korobeynikov A.; Pevzner P. A. metaSPAdes: a new versatile metagenomic assembler. Genome Res. 2017, 27, 824–834. 10.1101/gr.213959.116. 28298430 PMC5411777

[ref41] Hyatt D.; Chen G.-L.; LoCascio P. F.; Land M. L.; Larimer F. W.; Hauser L. J. Prodigal: prokaryotic gene recognition and translation initiation site identification. BMC Bioinform. 2010, 11, 1–11. 10.1186/1471-2105-11-119. PMC2848648 20211023

[ref42] Seemann T. Prokka: rapid prokaryotic genome annotation. Bioinformatics 2014, 30, 2068–2069. 10.1093/bioinformatics/btu153. 24642063

[ref43] Langmead B.; Salzberg S. L. Fast gapped-read alignment with Bowtie 2. Nat. Methods 2012, 9, 357–359. 10.1038/nmeth.1923. 22388286 PMC3322381

[ref44] Li H.; Handsaker B.; Wysoker A.; Fennell T.; Ruan J.; Homer N.; Marth G.; Abecasis G.; Durbin R.; The sequence alignment/map format and SAMtools. Bioinformatics 2009, 25, 2078–2079. 10.1093/bioinformatics/btp352. 19505943 PMC2723002

[ref45] Morris R. L.; Schmidt T. M. Shallow breathing: bacterial life at low O2. Nat. Rev. Microbiol. 2013, 11, 205–212. 10.1038/nrmicro2970. 23411864 PMC3969821

[ref46] Zhao Y.; Li M.-C.; Konaté M. M.; Chen L.; Das B.; Karlovich C.; Williams P. M.; Evrard Y. A.; Doroshow J. H.; McShane L. M. TPM, FPKM, or normalized counts? A comparative study of quantification measures for the analysis of RNA-seq data from the NCI patient-derived models repository. J. Transl. Med. 2021, 19, 1–15. 10.1186/s12967-021-02936-w. 34158060 PMC8220791

[ref47] Fish J. A.; Chai B.; Wang Q.; Sun Y.; Brown C. T.; Tiedje J. M.; Cole J. R. FunGene: the functional gene pipeline and repository. Front. Microbiol. 2013, 4, 291 10.3389/fmicb.2013.00291. 24101916 PMC3787254

[ref48] Edgar R. C.; Haas B. J.; Clemente J. C.; Quince C.; Knight R. UCHIME improves sensitivity and speed of chimera detection. Bioinformatics 2011, 27, 2194–2200. 10.1093/bioinformatics/btr381. 21700674 PMC3150044

[ref49] Wang Q.; Quensen J. F. III; Fish J. A.; Kwon Lee T.; Sun Y.; Tiedje J. M.; Cole J. R. Ecological patterns of *nifH* genes in four terrestrial climatic zones explored with targeted metagenomics using FrameBot, a new informatics tool. MBio 2013, 4, e00592–e00513. 10.1128/mBio.00592-13. 24045641 PMC3781835

[ref50] Wheeler T. J.; Eddy S. R. nhmmer: DNA homology search with profile HMMs. Bioinformatics 2013, 29, 2487–2489. 10.1093/bioinformatics/btt403. 23842809 PMC3777106

[ref51] Saitou N.; Nei M. The neighbor-joining method: a new method for reconstructing phylogenetic trees. Mol. Biol. Evol. 1987, 4, 406–425. 10.1093/oxfordjournals.molbev.a040454. 3447015

[ref52] Kopylova E.; Noé L.; Touzet H. SortMeRNA: fast and accurate filtering of ribosomal RNAs in metatranscriptomic data. Bioinformatics 2012, 28, 3211–3217. 10.1093/bioinformatics/bts611. 23071270

[ref53] Wang Q.; Garrity G. M.; Tiedje J. M.; Cole J. R. Naive Bayesian classifier for rapid assignment of rRNA sequences into the new bacterial taxonomy. Appl. Environ. Microbiol. 2007, 73, 5261–5267. 10.1128/AEM.00062-07. 17586664 PMC1950982

[ref54] Dolfing J. Protocols for calculating reaction kinetics and thermodynamics. In: Hydrocarbon and Lipid Microbiology Protocols (eds. McGenity T. J.; Timmis K. N.; Nogales B.) 155–163. (Springer: Berline, Heidelberg, 2015), 10.1007/978-3-662-45179-3.

[ref55] Ellis J. E.; Williams A. G.; Lloyd D. Oxygen consumption by ruminal microorganisms: protozoal and bacterial contributions. Appl. Environ. Microbiol. 1989, 55, 2583–2587. 10.1128/aem.55.10.2583-2587.1989. 2513776 PMC203126

[ref56] Paudyal S. Using rumination time to manage health and reproduction in dairy cattle: A review. Vet. Q. 2021, 41, 292–300. 10.1080/01652176.2021.1987581. 34586042 PMC8547861

[ref57] Magdalena J. A.; Angenent L. T.; Usack J. G. The Measurement, application, and effect of oxygen in microbial fermentations: Focusing on methane and carboxylate production. Ferment. 2022, 8, 138 10.3390/fermentation8040138.

[ref58] Li F.; Li C.; Chen Y.; Liu J.; Zhang C.; Irving B.; Fitzsimmons C.; Plastow G.; Guan L. L. Host genetics influence the rumen microbiota and heritable rumen microbial features associate with feed efficiency in cattle. Microbiome 2019, 7, 1–17. 10.1186/s40168-019-0699-1. 31196178 PMC6567441

[ref59] Kamke J.; Kittelmann S.; Soni P.; Li Y.; Tavendale M.; Ganesh S.; Janssen P. H.; Shi W.; Froula J.; Rubin E. M.; Attwood G. T. Rumen metagenome and metatranscriptome analyses of low methane yield sheep reveals a Sharpea-enriched microbiome characterised by lactic acid formation and utilisation. Microbiome 2016, 4, 1–16. 10.1186/s40168-016-0201-2. 27760570 PMC5069950

[ref60] Krieg N. R.; Ludwig W.; Euzéby J.; Whitman W. B. Phylum XIV. *Bacteroidetes phyl. nov**.* In: Bergey’s Manual of Systematic Bacteriology *Vol.* 4*:* *The Bacteroidetes, Spirochaetes, Tenericutes (Mollicutes), Acidobacteria, Fibrobacteres, Fusobacteria, Dictyoglomi, Gemmatimonadetes, Lentisphaerae, Verrucomicrobia, Chlamydiae, and Planctomycetes* (eds. Krieg N. R. et al.) 25–469 (Springer: New York, 2010), 10.1007/978-0-387-68572-4.

[ref61] Fu S. F.; Wang F.; Shi X. S.; Guo R. B. Impacts of microaeration on the anaerobic digestion of corn straw and the microbial community structure. Chem. Eng. J. 2016, 287, 523–528. 10.1016/j.cej.2015.11.070.

[ref62] Janssen P. H.; Kirs M. Structure of the archaeal community of the rumen. Appl. Environ. Microbiol. 2008, 74, 3619–3625. 10.1128/AEM.02812-07. 18424540 PMC2446570

[ref63] Ungerfeld E. M.; Kohn R. A. The role of thermodynamics in the control of ruminal fermentation. In: Ruminant physiology: digestion, metabolism and impact of nutrition on gene expression, immunology and stress (eds. Sejrsen K.; Hvelplund T.; Nielsen M.O.) 55–85 (Wageningen Academic Publishers, 2006).

[ref64] McCarty P. L.; Smith D. P. Anaerobic wastewater treatment. Environ. Sci. Technol. 1986, 20, 1200–1206. 10.1021/es00154a002.

[ref65] Joblin K. N. Ruminal acetogens and their potential to lower ruminant methane emissions. Aust. J. Agric. Res. 1999, 50, 1307–1314. 10.1071/AR99004.

[ref66] Hungate R. E. The rumen and its microbes (Elsevier, 2013).

[ref67] Beauchemin K. A.; Kreuzer M.; O’mara F.; McAllister T. A. Nutritional management for enteric methane abatement: a review. Aust. J. Exp. Agric. 2008, 48, 21–27. 10.1071/EA07199.

[ref68] Beauchemin K. A. Invited review: Current perspectives on eating and rumination activity in dairy cows. J. Dairy Sci. 2018, 101, 4762–4784. 10.3168/jds.2017-13706. 29627250

[ref69] Valente T. N. P.; Sampaio C. B.; Lima E. D. S.; Deminicis B. B.; Cezário A. S.; Santos W. B. R. D. Aspects of acidosis in ruminants with a focus on nutrition: a review. J. Agric. Sci. 2017, 9, 90 10.5539/jas.v9n3p90.

[ref70] Lim J. W.; Wang J.-Y. Enhanced hydrolysis and methane yield by applying microaeration pretreatment to the anaerobic co-digestion of brown water and food waste. Waste Manage. 2013, 33, 813–819. 10.1016/j.wasman.2012.11.013. 23290270

[ref71] Tsapekos P.; Kougias P. G.; Vasileiou S. A.; Lyberatos G.; Angelidaki I. Effect of micro-aeration and inoculum type on the biodegradation of lignocellulosic substrate. Bioresour. Technol. 2017, 225, 246–253. 10.1016/j.biortech.2016.11.081. 27898314

[ref72] Reeburgh W. S. Oceanic Methane Biogeochemistry. Chem. Rev. 2007, 107, 486–513. 10.1021/cr050362v. 17261072

[ref73] Angle J. C.; Morin T. H.; Solden L. M.; Narrowe A. B.; Smith G. J.; Borton M. A.; Rey-Sanchez C.; Daly R. A.; Mirfenderesgi G.; Hoyt D. W.; Riley W. J.; Miller C. S.; Bohrer G.; Wrighton K. C. Methanogenesis in oxygenated soils is a substantial fraction of wetland methane emissions. Nat. Commun. 2017, 8, 1–9. 10.1038/s41467-017-01753-4. 29146959 PMC5691036

