# Supplementary material for: Evaluation of Nanaerobic Digestion as a Mechanism to Explain Surplus Methane Production in Animal Rumina and Engineered Digesters
Source: Environ Sci Technol. 2023 Aug 11;57(33):12302–14. doi: 10.1021/acs.est.2c07813 (PMC10448717; doi:10.1021/acs.est.2c07813)
Supplement: Supplementary file 4 — es2c07813_si_004.pdf [file es2c07813_si_004.pdf]

Supporting Information for

Evaluation of nanaerobic digestion as a mechanism to explain surplus methane production in animal rumina and engineered digesters

Zhuoying Wu[a,e,*], Duc Nguyen[b,f], Shilva Shrestha[c,d], Lutgarde Raskin[c], Samir Kumar Khanal[b], Po-Heng Lee[a,*]

The supporting information includes five supplementary tables and one supplementary figure. While Tables S1 and S4 are shown in this Word file, Tables S2, S3 and S5 and Figure S1 are included in separate files.

Table S1 Bacterial oxidases that use O2 as terminal electron acceptor (provided in this Word file).

Table S2 Characteristics of the sequence datasets from animal rumina and engineered digesters (provided in a separate Excel file).

Table S3 Normalized abundance of genes or transcripts involved in nanaerobic digestion for each sample (provided in a separate Excel file). The file contains a total of 26 sheets, which includes a brief profile for all samples, and the detailed intermediate data for each sample. Some key sample information is shown in Table 1 in the main text.

Table S4 Thermodynamic conditions of animal rumina and ORP-controlled oxygenated anaerobic digesters (provided in this Word file).

Table S5 Thermodynamic calculations of animal rumina and ORP-controlled oxygenated anaerobic digesters (provided in a separate Excel file).

Figure S1 Phylogenetic tree constructed from 598 cytochrome bd oxidase amino acid sequences retrieved from all the samples (provided in a separate PDF file).

Table S1 Bacterial oxidases that use O_2 as terminal electron acceptor.

	**Low-affinity terminal oxidase**			**High-affinity terminal oxidase**		
Family	Alternative oxidase	Heme- and copper-containing terminal oxidases				Cytochrome *bd* oxidase
Class	AOX	A	A	B	C	Cyt *bd*
Enzyme	cyt bb_3	cyt aa_3 /cyt aa_3-600	cyt bo_3	cyt ba_3	cyt cbb_3	cyt *bd*
Catalytic subunit	*coxN*	*ctaD* /*qoxB*	*cyoB*	*cbaA*	*fixN* /*ccoN*	*cydA* /*appC* /*ythA*
O_2 affinity	18 μM	200 nM	150-350 nM	NA	40 nM	3-8 nM
Ref.	7	8	9	NA	10	11

NA stands for not available.

Table S4 Thermodynamic conditions of animal rumina and ORP-controlled oxygenated anaerobic digesters.

Thermodynamic Conditions		Rumen		ORP-AD
Components	Unit	High Roughage	High Concentrate	Replicate 1 -Day 149
O_2	atm	1.00E-07	1.00E-07	7.74E-10
H_2	atm	1.62E-03	2.63E-03	1.00E-05
CO_2	atm	0.679	0.760	0.432
CH_4	atm	0.334	0.253	0.508
Acetate	mM	70	67	8.40
pH	/	6.5	5.4	7
Ref.		63		17

High Roughage, animal fed with high roughage diet
High Concentrate, animal fed with high concentrate diet
AD, anaerobic digester
ORP-AD, ORP-controlled oxygenated anaerobic digester
Replicate 1-Day 149, condition at the day 149 of the first operation period
