# Supplementary material for: Evaluation of Nanaerobic Digestion as a Mechanism to Explain Surplus Methane Production in Animal Rumina and Engineered Digesters
Source: Environ Sci Technol. 2023 Aug 11;57(33):12302–14. doi: 10.1021/acs.est.2c07813 (PMC10448717; doi:10.1021/acs.est.2c07813)
Supplement: Supplementary file 5 — es2c07813_si_005.pdf [file es2c07813_si_005.pdf]

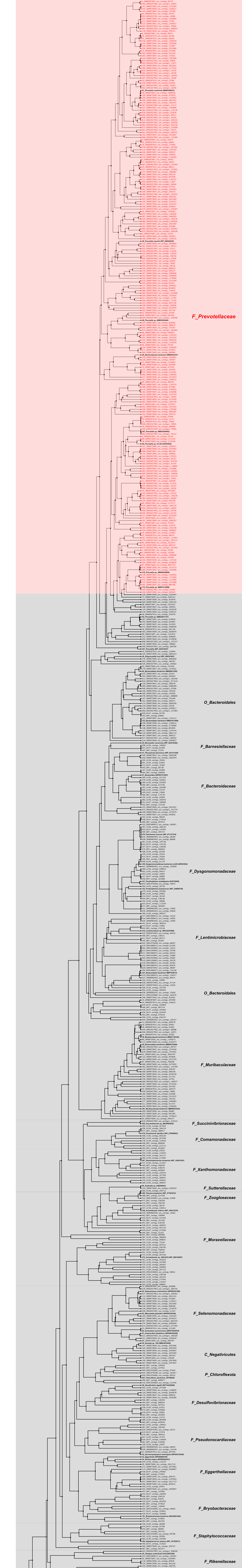

Fig S1. Phylogenetic tree constructed from 598 cytochrome bd oxidase amino acid sequences retrieved from all the samples. Sequenes from animal rumina are shown in red; it shows most ruminal sequences affiliated with the family *Prevotellaceae*. Meanwhile sequences from engineered digesters are shown in blue, it shows they are much different from the rumen populations. Specifically, the most abundant sequences (B147_contigs_19304 and A192_contigs_01176, Table S3) in the ORP-controlled digesters belonged to the genus *Proteiniphilum*, which was consistent with the results from our previous 16S rRNA gene and genome-based metagenomic analysis[17-18]. The sequence names for each sample are listed in Table S3 (excel file)."
